# Text Recognition Model Based on Multi-Scale Fusion CRNN

**DOI:** 10.3390/s23167034

**Published:** 2023-08-08

**Authors:** Le Zou, Zhihuang He, Kai Wang, Zhize Wu, Yifan Wang, Guanhong Zhang, Xiaofeng Wang

**Affiliations:** School of Artificial Intelligence and Big Data, Hefei University, Hefei 230601, China; zoule@mail.ustc.edu.cn (L.Z.);

**Keywords:** text recognition, feature fusion, multi-scale

## Abstract

Scene text recognition is a crucial area of research in computer vision. However, current mainstream scene text recognition models suffer from incomplete feature extraction due to the small downsampling scale used to extract features and obtain more features. This limitation hampers their ability to extract complete features of each character in the image, resulting in lower accuracy in the text recognition process. To address this issue, a novel text recognition model based on multi-scale fusion and the convolutional recurrent neural network (CRNN) has been proposed in this paper. The proposed model has a convolutional layer, a feature fusion layer, a recurrent layer, and a transcription layer. The convolutional layer uses two scales of feature extraction, which enables it to derive two distinct outputs for the input text image. The feature fusion layer fuses the different scales of features and forms a new feature. The recurrent layer learns contextual features from the input sequence of features. The transcription layer outputs the final result. The proposed model not only expands the recognition field but also learns more image features at different scales; thus, it extracts a more complete set of features and achieving better recognition of text. The results of experiments are then presented to demonstrate that the proposed model outperforms the CRNN model on text datasets, such as Street View Text, IIIT-5K, ICDAR2003, and ICDAR2013 scenes, in terms of text recognition accuracy.

## 1. Introduction

Scene text recognition involves recognizing a sequence of text with semantic information in a real-world scene, such as a billboard or street sign. The result is usually a text of varying length. The input images are gray, as text recognition does not need to take colour information into account. Scene text recognition has wide-ranging applications in computer vision, including intelligent robotics, autonomous driving, and other areas.

Traditional text recognition can only segment the text into individual characters, recognize the individual characters separately, and then switch the detected individual characters into a word by dynamic programming, lexicon search [1], and so on. The typical process for character recognition involves breaking it down into four stages: feature extraction, feature encoding, feature aggregation and feature classification. For feature extraction, one common approach involves extracting a multitude of local feature descriptors from an image at a fixed scale and step size. Popular methods used for this purpose include scale-invariant feature transform (SIFT) [2], local binary pattern (LBP) [3], histogram of oriented gradient (HOG) [4], and so on. Multiple features can also be used together to prevent losing too much useful information. However, the extracted features contain a lot of redundant and noisy information. In order to improve the robustness of the feature representation, the underlying features need to be encoded using a feature transformation algorithm. The classical feature coding methods include vector quantization [5], locality-constrained linear coding (LLC) [6], Fisher vector coding [7], and so on. Following feature encoding, spatial feature constraint (or “feature aggregation”) is applied by taking the average or maximum value of each dimensional feature within a spatial range. One common feature aggregation method, denoted as spatial pyramid pooling [8], involves dividing the image into blocks and applying a specific operation to each block, resulting in a fixed-dimensional vector after feature pooling. Finally, features are classified using a classifier, such as a support vector machine (SVM) [9], random forest [10], conditional random field [11] and so on. SVMs based on the kernel methods are the most widely used classifiers as they perform well on traditional image classification tasks; however, they are still somewhat less accurate than methods based on convolutional neural networks (CNNs).

CNN-based image recognition methods have successfully overcome the limitations of traditional approaches that require manual extraction of image features in many computer vision tasks. Convolutional neural networks, including LeNet-5 [12], AlexNet [13], VGG [14], and ResNet [15], have achieved great breakthroughs in the image recognition task. CNNs are trained by forward and backward propagation and gradient descent to automatically learn feature information in an image, which greatly improves the recognition accuracy of individual text. However, to recognize text sequences with contextual semantics, it is necessary to incorporate additional methods. For example, Wang et al. [1] proposed a method which combines individual text with dictionary search to recognize text sequences.

Text recognition models that combine CNN and a recurrent neural network (RNN) (e.g., CRNN [16], RARE [17], and other models) extract features from images and learn contextual semantic information by means of deep learning; thus, they significantly improve the accuracy of recognizing text with contextual semantics and have become the mainstream text recognition framework. The mainstream text recognition frameworks usually input images with text to a CNN to learn a set of feature sequences, then input the feature sequences to an RNN to learn contextual information, and finally output text according to the length of the feature sequences, thus, realizing text recognition for different lengths. However, when CNN learns image features, in order to obtain more feature sequences, the model usually uses a special downsampling method (e.g., 2 × 1 pooling), i.e., the height is reduced to half the original size, but the width remains the same. For example, the CRNN model uses four poolings in the convolutional layer by using VGG to extract image features. The first two poolings use a pooling size of 2 × 2, and the third and fourth pooling layers use a pooling size of 2 × 1, in order to obtain more feature sequences by pursuing a proper height to width ratio. However, the small perceptual field associated with this method does not allow for the extraction of features from the larger surrounding area, making the recognition of larger text ineffective. Therefore, a single downsampling method used in CNN is not conducive to better feature extraction.

This paper proposes a method to improve the accuracy of text recognition. The multiple scales fusion CRNN (MSF-CRNN) model incorporates multi-scale fusion into the CRNN model. The MSF-CRNN model uses different downsampling scales to obtain two different scales of feature output when extracting features in the convolutional layer. The feature sequences are then fed into the recurrent layer to learn the contextual information, and the final predicted results are output in the transcription layer.

The main contributions of the proposed MSF-CRNN model are outlined as follows:

(1) The proposed MSF-CRNN model uses a new multi-scale output CNN in the convolutional layer. The use of two different scales of downsampling when extracting image features allows for more image features and outputs two different scales of results.
(2) The MSF-CRNN model uses a new fusion approach in the feature fusion layer, which fuses the outputs of the different scales of the convolutional layer to better represent the features of the image and improve the accuracy of the recognized text.
(3) The proposed model was evaluated on datasets, such as IIIT5k [18], SVT [1], ICDAR2003 [19] and ICDAR2013 [20]. Quantitative and qualitative analyses were conducted, and the experimental results demonstrate that the accuracy of the MSF-CRNN model with multiscale fusion is higher than that of the model without multiscale fusion.

## 2. Related Work

In recent years, a large amount of scientific literature has been published on scene text recognition. A comprehensive survey can be found in the literature [21]. According to their implementation, text recognition algorithms are classified into traditional text recognition algorithms, text recognition algorithms based on attention mechanisms, and connectionist temporal classification (CTC).

Traditional text recognition algorithms rely heavily on manually crafted features, such as HOG and SIFT, to recognize text. These algorithms use techniques, such as hidden Markov models and SVM, to classify the text.

In contrast, attention-based text recognition algorithms dynamically select important parts of an image during recognition, reducing the reliance on manually engineered features. These algorithms use RNN combined with attention mechanisms to recognize text. Attention mechanisms enable these algorithms to focus on specific parts of an image while ignoring irrelevant regions, which improves recognition accuracy.

Text recognition algorithms based on CTC involve training a model to learn the probability distribution of output sequences and selecting the most likely output sequence for a given input image. These algorithms use deep neural networks, such as CNN or RNN, to extract features from the softmax layer to predict the output sequence.

Overall, while traditional text recognition algorithms have proven effective, attention- and CTC-based algorithms are becoming increasingly popular due to their ability to automatically learn features and achieve higher recognition accuracy.

### 2.1. Traditional Text Recognition Algorithms

Traditional text recognition methods follow a bottom-up approach that includes three steps: image preprocessing, character segmentation, and character recognition [21]. Individual characters are first detected using traditional methods, such as sliding windows [22], Hough voting [23], and connected components [24]. Dynamic programming and dictionary searching are utilized to form words from the individual characters that have been detected in the traditional text recognition method. There are also some top-down approaches to text recognition, where text is recognized directly from the entire input image, rather than detecting and recognizing individual characters. For example, the model proposed by Almázan et al. [25] takes the image after convolution and projects it into a subspace. The text recognition task has been modelled as a feature space word retrieval task. However, it is less accurate than CNN-based models. Jaderberg et al. [26] treated text recognition as a classification task. However, constructing a dataset with 90 k categories requires a large number of samples. Cheng et al. [27] proposed local monitoring of attention scores to mitigate the attention drift. The model proposed by Su and Lu et al. [28] first converts images into continuous HOG features and predicts the corresponding character sequences using an RNN. The model proposed by Ranjitha et al. [29] first segments the image by characters, extracts the required features using maximally stable extremal regions (MSERs), and then performs a stroke width transformation on the obtained results and merges all the processed regions to obtain accurate recognition results.

Due to the irregular arrangement of scene text and complex background, character segmentation is widely regarded as the most difficult task in achieving accurate recognition for the overall system. Character segmentation presents a considerable challenge to the recognition system’s performance. To overcome this issue, two main techniques are implemented, namely attention mechanisms and CTC [30]. Attention-based methods use RNN combined with attention mechanisms to focus on specific parts of an image, while CTC-based approaches involve training models to acquire a probability distribution over all potential output sequences, subsequently electing the most probable output sequence for a provided input image. These methods can recognize text without explicitly segmenting characters and achieve higher accuracy than traditional methods.

### 2.2. Text Recognition Algorithms Based on Attention Mechanisms

The use of attention mechanisms has been extended beyond machine translation systems and is now being implemented in scene text recognition with the aim of enhancing its performance. Bahdanau et al. proposed an attention mechanisms model [31] in 2014. Lee et al. [32] proposed an RNN for text recognition in wordless scenes. The model first extracted image features and then decoded the output characters using RNNs. However, the attention-based approach suffers from the problem of attention drift, so Cheng et al. [27] proposed localized supervision of attention scores to attenuate the attention drift. Bai et al. [33] found the problem of misalignment between attentional output sequences of probability distributions. A solution proposed by Bai et al. [33] is the “edit probability” (EP) metric. The EP model used maximum likelihood loss to reduce misalignment. Liu et al. [34] introduced an efficient attention-based encoder–decoder model. Some attention-based text recognition methods focus on recognition of irregular text recognition, such as Shi et al.’s [35] system that combines a spatial transformer network (STN) [36] with an attention-based text sequence recognition network. Cheng et al. [37] argue that encoding a text image as a one-dimensional feature sequence is insufficient and propose encoding the input image as a sequence of four features in four directions: horizontal, vertical, reverse, and horizontal. Liu et al. [38] introduced a hierarchical attention mechanism (HAM), which models the deformation of individual characters using local transformations, improving efficiency and handling different types of deformations difficult to model with a single global transformation. Liao et al. [39] transformed the recognition task into semantic slicing by treating each character type as a distinct class. This approach made it insensitive to shape, thereby rendering it effective for recognizing irregular text.

Another approach for recognizing curved text is the two-dimensional attention mechanism (2D attention) [40], which has been verified in the literature [41]. Qiao et al. proposed a semantically enhanced encoder–decoder framework [42] that exploits global semantic information to robustly identify low-quality scene text. This framework enhances the feature representation by incorporating both local and global contextual information, which improves recognition accuracy even under adverse conditions [43].

### 2.3. Text Recognition Algorithms Based on CTC

CTC has become a popular technique in scene text recognition. By using only sequence-level labels as supervision, this enables the network to be trained effectively. Graves et al. [44] proposed a CTC-based model for handwriting recognition. Since then, CTC has been applied to text recognition [45,46,47]. Shi et al. proposed the CRNN model [16], which combines RNN and CNN to recognize text images of scenes. The CRNN is composed of three components: a convolutional layer, a recurrent layer, and a CTC layer. Saffar et al. proposed to use the salp swarm optimization algorithm to optimize the parameters of convolutional neural network in DC-CRNN [48] to further improve the recognition accuracy of CRNN. The multi-modal text recognition network (MATRN) [49] proposed by Na et al. can better improve the accuracy of text recognition by fusing visual and semantic information. In addition, the parallel positional attention module (PPAM) [50] proposed by Fu et al. can improve the accuracy of recognition by parallel computing.

CLIP4STR [51], proposed by Zhao et al., is a simple and effective STR method for CLIP-based image and text encoders, which can effectively improve the accuracy of text recognition models. The PARSeq method [52] proposed by Bautista et al. uses permutation language modeling to learn an internal set of AR language models with shared weights and achieves good results on text datasets. The model proposed by He et al. [53] constructs a subgraph for each instance and trains it using graph convolutional network and cross-entropy loss function, which achieves good results in text recognition. Zheng et al. proposed a new multi-domain character distance perception (MDCDP) module [54] to establish visually and semantically relevant position encoding, so as to improve the recognition position of the model. Cui et al. proposed a representation and correlation enhanced encoder–decoder framework (RCEED) [55] to address these shortcomings and break through the performance bottleneck. In the encoder module, local visual features, global contextual features, and location information are aligned and fused to generate a small-sized comprehensive feature map. In the decoder module, two methods are used to enhance the correlation of the scene and text feature spaces.

In order to obtain better text recognition accuracy, we proposed a multi-scale fusion CRNN model. The CRNN model uses a combination of CNN and RNN, and we further consider the problem of multi-scale fusion by fusing different scales, both expanding the receptive field when extracting features and extracting more scales of text features, thus, improving the accuracy of text recognition.

## 3. Methodology

The overall framework of the MSF-CRNN model was proposed in Section 3.1. We proposed the convolutional layer of the MSF-CRNN model in Section 3.2. A multi-scale approach to feature extraction is proposed to address the problem that too small downsampling scale makes text recognition poor. That is, the features are extracted using different scales of downsampling to obtain two different scales of output. Different downsampling methods, namely MaxPooling and AvgPooling, are also used to extract features to address the problem that a single downsampling method cannot extract image features accurately.

The feature fusion layer of the MSF-CRNN model is developed in Section 3.3. Different fusion methods are compared in Section 3.3. The recurrent layer of the MSF-CRNN model is presented in Section 3.4. The application of a bidirectional RNN model to the task of contextual semantic learning in text is outlined. The loss function and the transcription layer are discussed in Section 3.5.

### 3.1. The Overall Model of the MSF-CRNN Model

**Figure 1:**
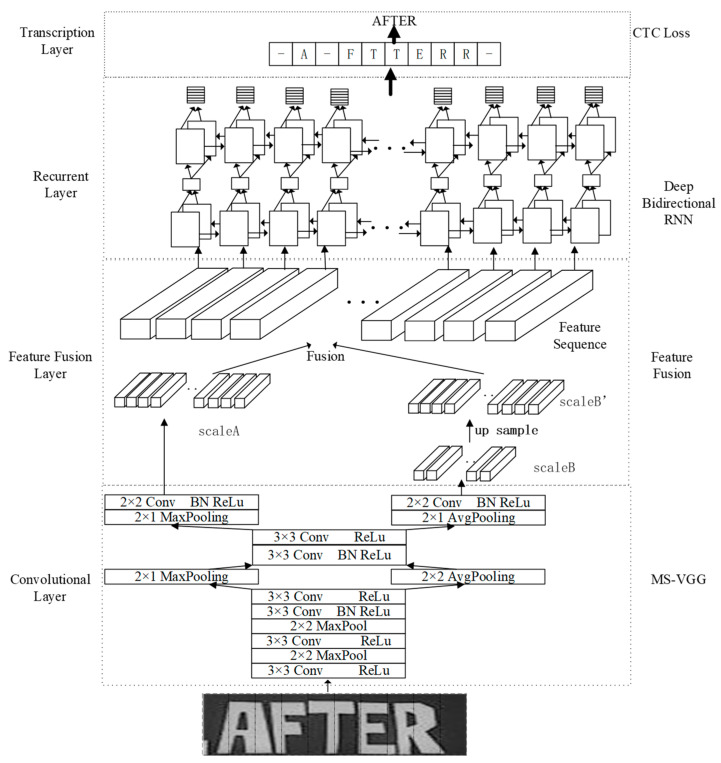
The framework of the MSF-CRNN model.

Figure 1 shows the overall structure of the MSF-CRNN model. The overall structure of the MSF-CRNN model has four layers, and our approach involves utilizing a multiple scales VGG (MS-VGG) convolutional neural network (CNN) architecture consisting of four main layers, namely the convolutional layer, the feature fusion layer, the recurrent layer, and the transcription layer. By applying this model to an input image with dimensions of 32 (height) × 40 (width) × 1 (gray-scale channel), we generate two feature sequences, scaleA and scaleB, through downsampling. Specifically, scaleA has a length of 10, while scaleB has a length of 5, resulting in different scales for the extracted features.

In the feature fusion layer, scaleB is upsampled to obtain scaleB’, and then scaleA and scaleB’ are fused to obtain a feature sequence of length 10. In the recurrent layer, a feature sequence of length 10 is fed to the RNN to learn the features of the context, and, for the RNN, an output result is obtained for each input. If the size of the input feature sequence is (1 × 10 × 512), then ten outputs are obtained. As shown in Figure 1, these ten outputs are -A-FTTERR-. In the transcription layer, the output of the recurrent layer is subjected to CTC to obtain the final result, which is the conversion of -A-FTTERR- to AFTER.

### 3.2. Convolutional Layer

In the convolutional layer of the MSF-CRNN model, we used different scales to extract the image features and obtained two different scales of features. Different downsampling methods are used to better extract the image features. The extraction of different scales and different downsampling methods will be described in the following sections.

#### 3.2.1. Extraction of Features at Different Scales

**Figure 2:**
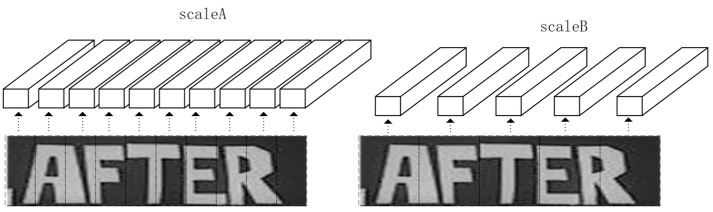
The first expressions of scaleA and scaleB.

When a CNN uses different downsampling scales, it obtains feature sequences of different lengths. The expressiveness of an image is different when it is downsampled to 1/32, 1/16, 1/8, 1/4. ScaleA means downsampling to 1/4, while scaleB means downsampling to 1/8 in Figure 2. If the image is downsampled to 1/4 of its original width, then the most significant number of feature sequences will be obtained, and smaller characters are displayed more clearly. However, there are also other problems: for some characters, the result of the downsampling may be the same, so it is not possible to identify the character exactly.

**Figure 3:**
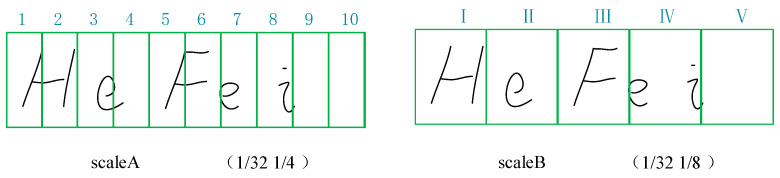
The second expressions of scaleA and scaleB 2.

As shown in Figure 3, (1/32, 1/4) of scaleA refers to downsampling the height size of the input image to 1/32 of its original size and reducing the width size to 1/4 of the original size. Similarly, for scaleB, (1/32, 1/8) means that the height size of the input image is reduced to 1/32 of the original size and the width size is reduced to 1/8 of the original size. The scaleA graph is capable of predicting ten characters, while the scaleB graph is capable of predicting only five characters.

It can be seen that the first block and the fifth block in scaleA are the same, so the same result is obtained after the CNN, but with two different letters, H and F. Even if the contextual features are learned through the recurrent layer, the accuracy of the recognition is still affected. In scaleB, it can be seen that in block IV, e and i are in a grid, and only one character can be recognized in a grid, but there are two characters. As such, the accuracy of the text recognition was affected.

**Figure 4:**
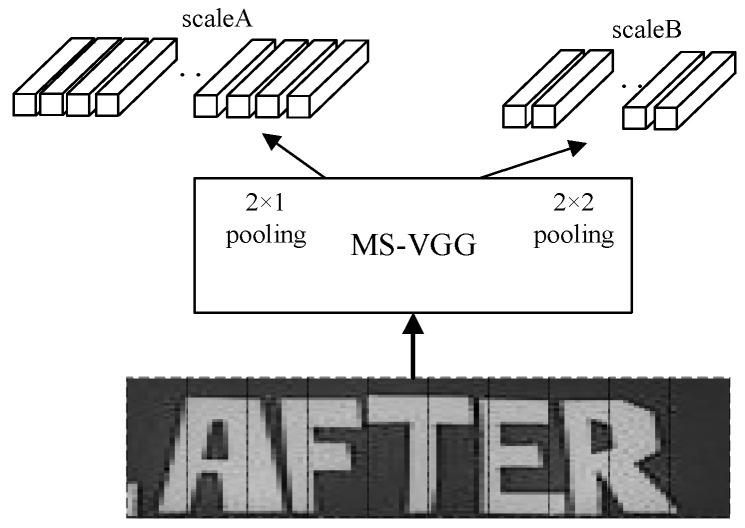
Output of two different scales in the convolution layer.

In order to better extract the information of the text, multiple scales VGG (MS-VGG) in the proposed MSF-CRNN model obtains two different scales by different downsampling. Here, 2 × 1 pooling is used for scaleA and 2 × 2 pooling is used for scaleB. The features of the smaller text are extracted in scaleA and the features of the larger text are extracted in scaleB. The results of the two different scales are shown in Figure 4.

#### 3.2.2. Different Downsampling Methods

**Figure 5:**
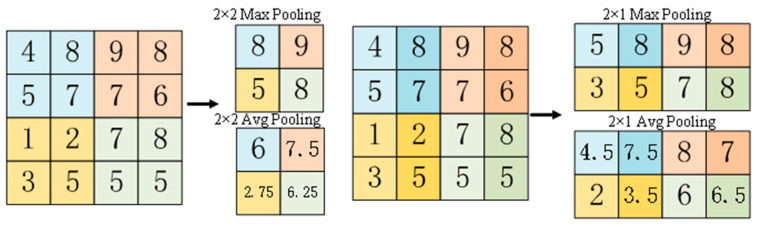
MaxPooling and AvgPooling.

The common ways of downsampling in convolutional neural networks are MaxPooling and AvgPooling. We present the implementation of AvgPooling and MaxPooling in Figure 5. MaxPooling retains the most “important” features in the local region when downsampling and uses the important features to determine the class of the image. AvgPooling selects the average features in the region when downsampling and reflects the global characteristics of the region more clearly.

**Figure 6:**
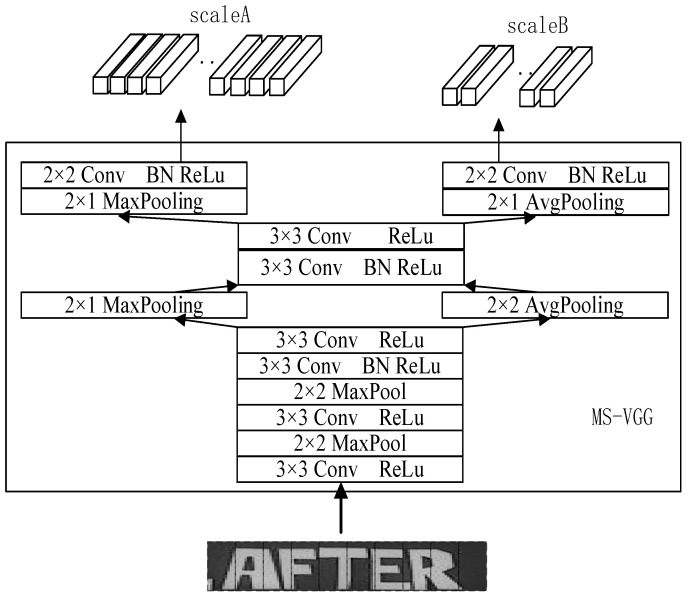
Extraction of features using different downsampling methods in MS-VGG.

To better learn the features of the image, a combination of MaxPooling and AvgPooling is used in multiple scales VGG (MS-VGG). For scaleA, MaxPooling is used to downsample and derive the most important features in the region, while for scaleB, AvgPooling is used to downsample, and the average features of the whole region were derived. The general structure of MS-VGG is shown in Figure 6. The MS-VGG model is divided into two branches: scaleA uses MaxPooling and scaleB uses AvgPooling. To speed up the training, we use a common convolutional layer in the MS-VGG model.

### 3.3. Feature Fusion Layer

In the feature fusion layer, in order to fuse two features of different scales (scaleA and scaleB) and better represent the features of the image, scaleB’ is obtained by upsampling scaleB so that scaleA and scaleB’ have the same dimensions. Then, we can fuse the scaleA and scaleB’ features.

#### 3.3.1. Upsampling of ScaleB

**Figure 7:**
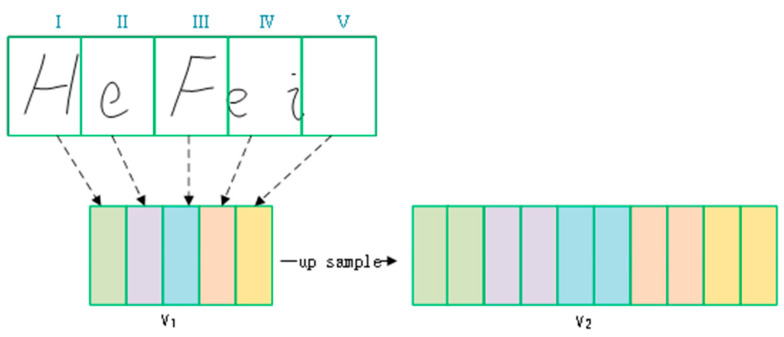
Upsampling method for the feature sequence.

In order to fuse scaleA and scaleB, scaleB needs to be upsampled to obtain scaleB’, which is shown in Figure 7.

Let the original feature sequences be $V_{1}$, and we obtain $V_{2}$ after up sampling. Let the dimensions of $V_{1}$ be [*l*, ω, *c*], then the dimensions of $V_{2}$ be [*l*, 2ω, *c*], where *l* represents height, ω represents width, and c denotes the number of channels, $V_{2}$ is obtained from Equation (1).

(1) $$V_{2}[l,i,c]=\left\{\begin{array}{c} V_{1}[l\;,\;\frac{i}{2}\;,\;\;c]\;\;\;\;\mathrm{if}\;i\;\mathrm{is}\;\mathrm{even}\\ V_{1}[l,\;\frac{i+1}{2},c]\;\;\;\mathrm{if}\;i\;\mathrm{is}\;\mathrm{odd} \end{array}\right.\;\;\;\;1\;\leq \;i\;\leq \;2\omega \;$$

#### 3.3.2. Fusion of scaleA and scaleB

**Figure 8:**
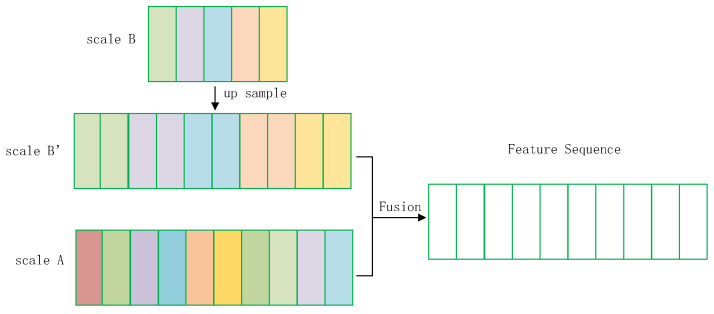
Fusion of scaleA and scaleB.

Figure 8 shows the fusion of scaleA and scaleB’. There are two ways to merge: concat and add. The concat method superimposes the two results to obtain a number of channels of 2 × *c*. For example, scaleA has dimensions [1, 10, 512] and scaleB has dimensions [1, 10, 512], which are fused to obtain [1, 10, 1024]. Since the number of channels input to the recurrent layer is set to 512, it is necessary to change the number of channels from 1024 to 512 by 1 × 1 convolution. This will increase the training time.

The add method involves adding the corresponding elements, for example, the size of scaleA is [1, 10, 512], the size of scaleB is [1, 10, 512]; by adding the corresponding elements, the result is still [1, 10, 512], which can be sent directly to the recurrent layer to learn the context information. The accuracy of the concat and add methods was compared in experiment 2 in the experiments section. Since the results obtained by concat have to undergo a 1 × 1 convolution, which increases both the training time and the training difficulty, the MSF-CRNN model was fused using the add method.

### 3.4. The Recurrent Layer

To better learn the contextual information, we use a bi-directional RNN, so that the network learns both the information of the previous text and the later text. There are two mainstream approaches to bidirectional recurrent neural networks (bi-directional RNNs). The first is the BiLSTM [56], which utilizes a complex architecture consisting of memory cells and gates to selectively regulate information flow between time steps in both forward and backward directions. The second model is the BiGRU [57], which employs gating mechanisms to control the amount of information that is retained or discarded at each time step, allowing for efficient processing of long sequences while avoiding the vanishing gradient problem. To verify the reliability of the two models, BiLSTM and BiGRU, were used in the experimental part. The results show that BiLSTM is more accurate, but requires more model parameters, while BiGRU is less accurate, but uses fewer model parameters. To achieve higher accuracy, the MSF-CRNN model uses Bi-LSTM as the recurrent layer of the bi-directional RNN.

### 3.5. The Transcript Layer and Loss Function

#### 3.5.1. The Transcript Layer

**Figure 9:**
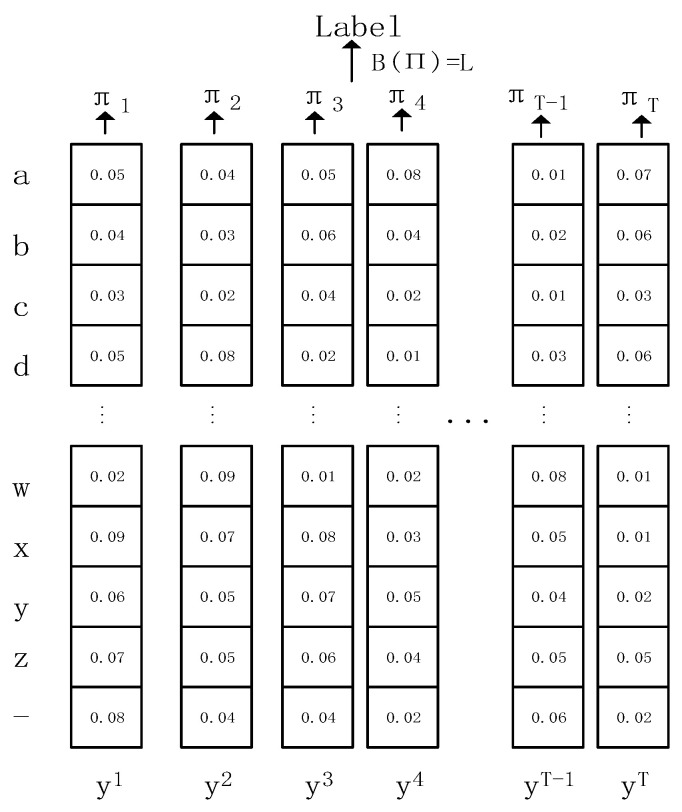
Output of the recurrent layer.

The transcription layer uses the conditional probabilities defined by the CTC to convert the predicted results of the recurrent layer into a sequence of labels. Figure 9 shows the output of each frame from the recurrent layer. The output of each frame is a vector representing the probability of being a particular character. The transcription layer finds the sequence of labels with the highest probability given the conditions predicted for each frame. In practice, when it comes to speech recognition and transcription, two models are often used in speech recognition and transcription: lexicon-free transcription and lexicon-based transcription. Lexicon-based transcription means that all possible words are predetermined, and then the output is matched to the words in the lexicon. However, the model proposed in this paper can predict any word, so there is no predetermined lexicon.

Let the output of the recurrent layer be $y=y^{1},y^{2},y^{3},...,y^{T}$, where T denotes the length of the feature sequence, $y^{i}$ is a vector with length *n*, $y^{i}=(y_{1}^{i},y_{2}^{i},y_{3}^{i},...,y_{n}^{i})$, *n* denotes the number of characters to be predicted plus one blank character (if only lowercase letters are predicted, then n = 26 + 1 = 27), and $y_{k}^{i}$ denotes the probability that the vector $y^{i}$ will predict the kth character, so $\sum_{k}y_{k}^{i}=1$.

Define the mapping function B with input $\pi =(\pi_{1},\pi_{2},\pi_{3},...,\pi_{T})$, and the output is L, i.e., B(π)=L.  The mapping function B performs the following two steps in sequence: (1) removing consecutive repetitive characters and (2) removing blank characters.

As an example: B(--hh-e-l-ll-oo--) = hello, B(-hhh-eel-llloo--) = hello.

P(π|y) denotes the probability of obtaining π conditional on input *y*, which is calculated as shown in Equation (2), and $y_{\pi_{i}}^{i}$ denotes the probability that the *i*-th output is predicted to be character $\pi_{i}$, as follows:

(2) $$P(\pi |y)=\prod_{i=1}^{t}y_{\pi_{i}}^{i}$$

$P\left(L|y\right)$ represents the probability of obtaining the label *L* given the input *y*, which is calculated as shown in the following Equation (3):

(3) $$P(L|y)=\sum_{\pi :B(\pi )=L}P(\pi |y)$$

where P(L|y) takes the maximum value for π, and $I^{*}=B(\pi )$ is used as the predicted result. However, it would take a lot of time to use the exact finding method, and to speed up the finding, a fuzzy finding strategy is used, under which $I^{*}\approx B({argmax}_{\pi }P(\pi |y))$, i.e., each P(π|y) outputs only the maximum probability.

#### 3.5.2. The Loss Function

This paper sets the loss function *O* as a negative log-likelihood function of the conditional probability and trains the model by minimizing this loss function. This approach is commonly used in machine learning, where the goal is to train a model that can accurately predict outputs given inputs. The negative log-likelihood function measures the difference between the predicted probabilities and the actual outcomes and penalizes incorrect predictions more heavily. Equation (4) is given as follows:

(4) $$O=-\sum_{I_{i},L_{i}}\log P(L_{i}|y_{i})$$

where $X=\{I_{i},L_{i}\}$ refers to the training set, $I_{i}$ represents the training image, $L_{i}$ represents the real label sequence, and $y_{i}$ represents the output of the recurrent layer.

## 4. Experimental Results

The MSF-CRNN model was trained and tested on a desktop computer with an Intel i5-9500 CPU (3.00 GHz), 32 GB RAM, and a GTX 1080 Ti 11 GB, using Python 3.7. The code in this paper uses the PyTorch 1.8 deep learning framework, where the input images are scaled equally and the height is uniformly modified to 32, the learning rate is 3 × 10^−3^, and the gradient descent method is Adam. The model utilizes an exponential decay rate of 0.5 to estimate the first-order moments, and a decay rate of 0.999 to estimate the second-order moments.

As the text recognition is different from image classification, in order to better evaluate the effectiveness of the model recognition, two metrics are used for evaluation in this paper, namely the average precision (AP) and the average precision of character (APc).

### 4.1. Benchmark Dataset

To evaluate the effectiveness of the proposed scene text recognition algorithm, we have employed four widely recognized and commonly used datasets in the field, namely ICDAR2003 (IC03), IIIT 5k-word (IIIT5k), ICDAR2013 (IC13), and Street View Text (SVT). By utilizing these diverse datasets, we hope to provide a comprehensive evaluation of the proposed algorithm’s effectiveness under different conditions and scenarios.

The ICDAR2003 dataset [19] consists of 251 images of scenes with labels. We cropped out images that contained only characters of letters or numbers and had more than or equal to three characters. The dataset contains 1823 character images, with 936 assigned to the training set and 860 to the test set.

The ICDAR2013 dataset [20] consists of 462 (training 229, test 233) images of natural scenes annotated in English. We discarded images that contained only characters of numbers or letters and when the number of characters was more significant than or equal to 3, resulting in 680 training images and 857 test images.

The IIIT 5k-word dataset [18] contains 3000 cropped word test images, which we cropped out to contain only numbers or letters, resulting in 2000 training images and 3000 test images.

The SVT dataset [1,22] contains 249 street view images. We cropped out images containing only characters with numbers or letters, resulting in 647 training images and 257 test images.

### 4.2. Model Performance Comparison

#### 4.2.1. Qualitative Comparison

**Figure 10:**
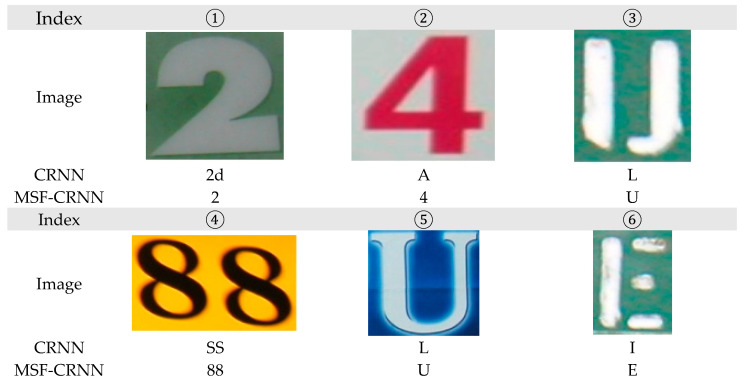
The MSF-CRNN and CRNN model recognition results.

In this section, we give an experiment to show the prediction results of the CRNN model with and without multi-scale fusion. Figure 10 shows the recognition results of some images after the MSF-CRNN and CRNN models, respectively. It can be seen that the CRNN model uses a too small downsampling scale in order to obtain more feature sequences, and the recognition cannot fully extract the whole text information completely, which leads to the decrease in recognition accuracy. The MSF-CRNN model incorporates multi-scale fusion to obtain results at different scales by downsampling at different scales and fusing the results at different scales, which enables the MSF-CRNN to predict larger text more accurately.

From Figure 10, in the first image, we can see that the CRNN model identifies a “2d”, but the other half is incorrectly identified as “d” due to the small receptive field, whereas the MSF-CRNN model more correctly identified the text as “2” due to the multi-scale fusion. In the second image, the CRNN extracts only half of the features of the number “4” due to the small receptive field and identifies it as “A”. The MSF-CRNN model, on the other hand, fuses the features of different scales to extract more comprehensive information and correctly identifies it as “4”. The CRNN is also incorrectly identified because of the too small receptive field, whereas the MSF-CRNN model is able to recognize the exact text content in other images in Figure 10.

#### 4.2.2. Quantitative Comparison

The proposed MSF-CRNN model is a complex and sophisticated approach to scene text recognition, which requires careful validation to demonstrate its effectiveness. Therefore, to evaluate the validity of this model, the authors have conducted three separate experiments in this paper. The first experiment compares the different effects of different scales for the feature scales in part (1) of Section 3.2. The second experiment compares the recognition accuracy of the two fusion methods, namely add and concat, respectively, in Section 3.3. The third experiment demonstrates that the MSF-CRNN model, which incorporates multi-scale fusion has a higher text recognition accuracy than the CRNN model. To further validate the reliability of the model, experiment 3 also uses BiLSTM and BiGRU in the recurrent layer for comparison, respectively.

In the first experiment, we give the comparison results of the effects with different downsampling. To verify the effect of features at different scales, we obtain the results after downsampling at different scales on the ICDAR2003 dataset by using the CRNN model. Here, S4, S8, S16 represent the accuracy of downsampling to the original 1/4, 1/8, 1/16, respectively, where S4 corresponds to the scaleA and S8 corresponds to scaleB in Section 3.2.

Table 1 defines the meaning of S4, S8, and S16 in the context of the input size. Specifically, S4 refers to scanning the input size down to 1/4 of its original size, S8 means scanning it down to 1/8 of its original size, and S16 means scanning it down to 1/16 of its original size.

Table 1 shows that different downsampling scales have different effects, with S4 being the best, followed by S8, and S16 being the worst. This is mainly because the downsampling scale of S16 is too large, resulting in only one result for multiple characters, which reduces the accuracy rate. S4 corresponds to the scaleA scale and S8 corresponds to the scaleB scale. It can be seen from the experiment that the difference between the accuracy of S4 and S8 is not large, but the difference between the accuracy of S8 and S16 is large. Both S4 and S8 scales can extract the features of the image relatively well, so the model proposed in this paper complements their advantages by combining S4 and S8 to obtain better text recognition results.

To provide a comprehensive comparison of the effectiveness of different fusion methods, this paper conducts an experiment to evaluate their performance. In order to compare the two fusion methods, namely add and concat, in Section 3.3, the MSF-CRNN model was used on the ICDAR2003 dataset to obtain the accuracy rates by using two different fusion methods, respectively.

From Table 2, after analyzing the results of the experiment, it is clear that the add fusion method outperforms the concat method in terms of accuracy. Specifically, the accuracy obtained by the add fusion method was found to be higher than that of the concat method. There are three reasons for this. Firstly, the concat fusion method has to change the number of channels of the image by 1 × 1 convolution, which makes training difficult and increases the training time and makes it difficult to converge to good results. Secondly, the concat fusion method is a superposition of two features, which does not fuse the features of two scales well. Thirdly, the add fusion method adds the elements corresponding to the two features, which can fuse the features of two different scales well. The computation is simpler, and the training is relatively easy. Therefore, the add fusion method is used for fusion in the MSF-CRNN model.

To further validate the reliability of the MSF-CRNN and CRNN models, in the third experiment, we use BiGRU and BiLSTM in the convolutional layer to learn contextual information, respectively. The two models were tested on the ICDAR2003, ICDAR2013, IIIT5K, and SVT datasets, respectively, and the results obtained are shown in Table 3 and Table 4. Table 3 shows the results obtained by comparing the performance of the proposed MSF-CRNN model against the CRNN model, when BiGRU is used in the convolutional layer. The evaluation is carried out on different benchmark datasets to assess the effectiveness of the models under different conditions and scenarios.

The experimental results demonstrate that the proposed MSF-CRNN model can also improve the accuracy of recognizing scene text compared to the CRNN model when using BiGRU and BiLSTM in the recurrent layer.

It can be seen from Table 4 that the accuracy of the MSF-CRNN model with multi-scale fusion on the ICDAR2003, ICDAR2013, IIIT5K, and SVT datasets is increased by 5.9%, 8.2%, 8.1%, and 7.3%, respectively, and the model parameters of MSF-CRNN and CRNN are almost unchanged. Experimental results show that the model with multi-scale fusion has higher recognition accuracy than the model without fusion.

The use of different RNNs in the recurrent layer shows that different RNNs have their own advantages. After analyzing Table 3 and Table 4, it is clear that using BiLSTM for the recurrent layer results in higher recognition accuracy compared to using GRU. However, the disadvantage of this approach is that it requires a significantly larger number of parameters. When BiGRU is used, the recognition accuracy is slightly lower, but the number of parameters required is also lower. Therefore, different RNNs can be used according to different needs. When using the same recurrent neural networks in the convolutional layer, the MSF-CRNN model can perform better than the model without multi-scale fusion.

There are three reasons for improving the accuracy of text recognition, as follows: (1) The MSF-CRNN model expands the receptive field when extracting image features, allowing a larger range of features to be seen; (2) the use of two different downsampling methods to extract more image features, MaxPooling for smaller scales and AvgPooling for larger scales, enables a more comprehensive extraction of image features and makes it easier to distinguish between different types of characters; (3) the fusion method of add can fuse the features of two different scales well, and it is easier to train the network after using add than other fusion methods, so that good recognition results can be achieved more quickly.

As shown in Table 5, the inference time is the time required to predict each image in ms. The training time is the time needed for each epoch. It can be seen that the inference time and training time of the multi-scale fusion model are slightly higher than that of the model without multi-scale, but the recognition accuracy of the model is significantly improved. In general, compared with the model without fusion, the multi-scale fusion model consumes more time but achieves a more significant improvement in accuracy.

To evaluate the recognition effect of the MSF-CRNN model, we compare it with other mainstream models and analyze their performance in terms of recognition accuracy. These models include R2AM [32], RARE [17], FAN [27], NRTR [58], Char-Net [38], ASTER [35], MORAN [59], SCATTER [60], and RCEED [55]. The models were published between the years 2016 to 2022, and their performance results are summarized in Table 6. By comparing the tabular data, we can derive a comprehensive assessment of the recognition accuracy of different models. The purpose of this comparison is to help us understand the relative performance of the MSF-CRNN model in competition with other popular models.

Based on the information provided and the data in Table 6, we can draw some conclusions. Firstly, the accuracy of MSF-CRNN on the four datasets ICDAR2003, ICDAR2013, IIIT5K, and SVT is 96.3%, 94.9%, 91.3%, and 90.1%, respectively. It is particularly noteworthy that MSF-CRNN achieves the highest accuracy on the ICDAR2013 dataset. In addition, the accuracy on other datasets is also close to the performance of other models. By fusing information from different scales, the MSF-CRNN model is able to extract more useful information from the data. This fusion process significantly enhances the recognition accuracy of the model. The ability to leverage multi-scale information enables the model to better handle text images with varying scales and features, leading to improved overall performance. In summary, the MSF-CRNN model demonstrates high accuracy in text recognition tasks, particularly on the ICDAR2013 dataset. Its ability to fuse multi-scale information allows it to effectively utilize features from different scales and adapt to diverse text images, resulting in improved recognition performance across multiple datasets.

## 5. Conclusions

The existing text recognition models have too small downsampling scale to better extract features from images, so we propose a new text recognition model that incorporates multi-scale fusion. The MSF-CRNN model is divided into a convolutional layer, a feature fusion layer, a recurrent layer, and a transcription layer. In the convolutional layer, two different scales of features, scaleA and scaleB, are obtained by convolutional neural networks. In the feature fusion layer, scaleB is upsampled and fused with scaleA to obtain a new feature sequence, and then the feature sequence is fed into the recurrent layer to learn the contextual information. We use Bi-LSTM in the recurrent layer. Finally, the final result is output through the transcription layer. The experimental results show that the proposed MSF-CRNN model has better performance on four publicly available text datasets.

In future research, different fusion methods will be considered to better extract the features of the image, so as to further improve the accuracy of text recognition. In addition, the model will be appropriately lightweight for easy deployment, and we will also consider using different convolution methods and transformer methods to extract image features.

## Figures and Tables

**Table 1:** Recognition precision of feature sequences extracted with different scales.

The Way of Scales	Average Precision (AP)	Average Precision of Character (APc)
S4	86.3	97.4
S8	65.3	82.1
S16	45.3	74.0

**Table 2:** Comparison of the accuracy of different fusion methods.

Model	Average Precision (AP)	Average Precision of Character (APc)
MSF-CRNN + concat	31.1	78.3
MSF-CRNN + add	80.4	91.4

**Table 3:** Results of the MSF-CRNN model and the CRNN model by using BiGRU on different datasets.

Models	Weights	ICDAR2003	ICDAR2013	IIIT5K	SVT
CRNN (BiGRU)	29.3M	87.3	84.2	80.1	79.1
MSF-CRNN (BiGRU)	31.3M	91.2	92.9	83.3	83.5
		+3.9	+8.7	+3.2	+4.4

**Table 4:** Results of the MSF-CRNN model and the CRNN model by using BiLSTM on different datasets.

Models	Weights	ICDAR2003	ICDAR2013	IIIT5K	SVT
CRNN (BiLSTM)	31.8M	90.4	86.7	83.2	82.8
MSF-CRNN (BiLSTM)	33.9M	96.3	94.9	91.3	90.1
		+5.9	+8.2	+8.1	+7.3

**Table 5:** Model training time and inference time.

Models	Inference Time	Training Time (min/epoch)			
		ICDAR2003	ICDAR2013	IIIT5K	SVT
CRNN (BiGRU)	42 ms	20 min	30 min	18 min	17 min
MSF-CRNN (BiGRU)	46 ms	22 min	32 min	20 min	19 min
CRNN (BiLSTM)	53 ms	25 min	35 min	23 min	22 min
MSF-CRNN (BiLSTM)	58 ms	26 min	36 min	23 min	24 min

**Table 6:** Comparison of MSF-CRNN with other models.

Models	ICDAR2003	ICDAR2013	IIIT5K	SVT
R2AM (2016) [32]	97.0	90	78.4	80.7
RARE (2016) [17]	90.1	88.6	81.9	81.9
FAN (2017) [27]	94.2	93.3	87.4	85.9
NRTR (2017) [58]	95.4	94.7	86.5	88.3
Char-Net (2018) [38]	93.3	91.1	92	87.6
ASTER (2018) [35]	94.5	91.8	93.4	89.5
MORAN (2019) [59]	95.0	92.4	91.2	88.3
SCATTER (2020) [60]	96.1	93.8	92.9	89.2
RCEED (2022) [55]	-	94.7	94.9	91.8
MSF-CRNN (ours)	96.3	94.9	91.3	90.1

## Data Availability

The data that support the findings of this study are openly available in reference number [1,18,19,20].
